# Efficacy of CDK9 inhibition in therapy of post-myeloproliferative neoplasm (MPN) secondary (s) AML cells

**DOI:** 10.1038/s41408-022-00618-4

**Published:** 2022-01-31

**Authors:** Warren Fiskus, Taghi Manshouri, Christine Birdwell, Christopher P. Mill, Lucia Masarova, Prithviraj Bose, Tapan M. Kadia, Naval Daver, Courtney D. DiNardo, Gautam Borthakur, Joseph D. Khoury, Srdan Verstovsek, Kapil N. Bhalla

**Affiliations:** The University of Texas M.D. Anderson Cancer Center, Houston, TX USA

**Keywords:** Acute myeloid leukaemia, Targeted therapies

**Dear Editor**,

Signaling downstream of the pathogenetic mutations in JAK2, MPL, or calreticulin, along with co-occurring mutations in chromatin modifiers or transcription factors, results in dysregulated transcriptome and proteome that is responsible for the transformation of myeloproliferative neoplasms (MPN) to sAML, as well and for conferring therapy refractoriness [1, 2]. Although routinely used and effective in symptomatic and advanced MPN, JAK inhibitor (JAKi), e.g., ruxolitinib, and/or treatments with standard AML chemotherapy are ineffective against post-myeloproliferative neoplasm (MPN) sAML [3]. Binding and activity of transcription factors (TFs), including lineage-specific master regulators and signaling TFs such as STAT3/5, RELA, and MYC, at enhancers and promoters of their targets involves recruitment of transcriptional co-factors and epigenetic regulators, including HAT (histone acetyltransferase), bromodomain extra-terminal (BET) protein (BETP) BRD4 and pTEFb (positive transcript elongation factor b) [4]. Collectively, activities of these TFs and co-factors induce promoter-proximal pause-release of the poised RNA pol II (RNAP2) to stimulate productive mRNA transcript elongation, leading to the dysregulated transcriptome that confers the aggressive phenotype and therapy refractoriness in post-MPN sAML cells [2, 5]. The non-cell-cycle regulatory CDK9 is the catalytic subunit of the positive transcript elongation factor b (pTEFb) [5, 6]. In association with its regulatory subunit cyclin T1, CDK9 mediates phosphorylation of serine 2 in the tandem heptad repeats of the C-terminal domain of RNA pol II (RNAP2), which induces pause-release of RNAP2. CDK9 also phosphorylates and inactivates the negative transcription regulators NELF (negative elongation factor) and DSIF (DRB sensitivity-inducing factor), thereby further promoting RNAP2-mediated transcription [5, 6]. CDK9 activity is required to maintain constant production of mRNAs of short-lived proteins, e.g., MCL1 and c-Myc, promoting growth and survival of AML cells [6, 7]. Dysregulated c-Myc in AML cells, either due to amplification or protein stabilization, interacts with and recruits pTEFb to its own enhancers and promoter and those of its target genes to mediate RNAP2 pause-release [6, 7]. Of the two protein isoforms of CDK9, the smaller 42 kDa isoform is more abundant in AML cells [6]. Selective CDK9 inhibitors that exhibit high affinity interaction with the ATP binding site of CDK9 inhibit phosphorylation of serine 2 of RNAP2, and CDK9-mediated phosphorylation of the negative regulators of RNAP2-mediated transcription, i.e., NELF and DSIF, thus inhibiting RNAP2-mediated transcription of oncogenes, e.g., c-Myc and MCL-1 [5–7].

In the present studies, we determined effects of two chemically distinct CDK9 inhibitors, BAY-1143572 and NVP2, on the post-MPN sAML cell lines SET-2 and HEL92.1.7 (HEL) and on primary patient-derived (PD) post-MPN sAML cells [8, 9]. Genetic alterations in the cell lines and PD post-MPN sAML cells are presented in Fig. S1A. As shown, NVP2 was more potent than BAY-1143572 to dose-dependently induce apoptosis in SET-2 compared to HEL cells (Fig. 1A). NVP2 and BAY-1143572 also exerted similar levels of lethal activity against the previously reported, in vitro isolated and characterized, ruxolitinib-persister (tolerant)/resistant (P/R) post-MPN sAML SET-2-RuxP and HEL-RuxP cells, as compared to the parental SET-2 and HEL92.1.7 cells (Fig. 1B and S1B) [10]. SET-2-RuxP and HEL-RuxP cells had been shown to exhibit non-genetic resistance to ruxolitinib, with LD_50_ values over 2000 nM in RuxP cells compared to 560 nM and 1350 nM for parental SET-2 and HEL92.1.7 cells (Fig. S1C), and cross-resistance to other JAKi [10]. Treatment with NVP2 and BAY-1143572 also dose-dependently induced in vitro loss of viability in multiple samples of PD, CD34+ sAML cells, harvested from patients who had been previously treated with ruxolitinib (Fig. 1C). In contrast, exposure to similar doses and exposure interval to BAY-1143572 or NVP2 did not induce loss of viability in CD34+ normal progenitor cells (Fig. S1D). We also determined effects of the CDK9is on the chromatin and transcriptome of post-MPN sAML cells. Following treatment of SET-2 cells with BAY-1143572 or NVP2, assessment of the accessible chromatin by ATAC-Seq demonstrated large numbers of lost and gained peaks (Fig. 1D) [11]. Rank-sorted TF motifs lost from the chromatin following treatment with BAY-1143572 or NVP2 included those of ERG, PU.1, RUNX1, STAT3/5, and c-Myc (Fig. S2A). Additionally, treatment with BAY-1143572 or NVP2 significantly reduced ATAC-Seq peaks over the super-enhancers of MYC, BCL2, and CDK6 (Fig. 1E, S2B, C). A previously reported RNA-Seq analysis had shown that NVP2 treatment repressed vastly greater numbers of mRNA expressions in leukemia cells, with log2 fold-reduction in the mRNAs of MYC, PIM1, MYB, LMO2, NFkB2, MCL1, BIRC3, BCL2L1, BCL2, and CDK6 (Fig. S2D, E) [9]. Utilizing qPCR analysis, we confirmed that NVP2 treatment repressed these same mRNAs, and of SPI1, RELB, PRDM1, and cFLIP in SET-2 and HEL cells (Fig. 1F and S3A). It is noteworthy, that treatment with NVP2, without affecting protein levels for the 42 kDa protein isoform of CDK9, also attenuated protein levels of cyclin T1, p-Rbp1 subunit of RNAP2, c-Myc, XIAP, and MCL1 in SET-2 and HEL92.1.7, as well as in SET-2-RuxP cells (Fig. 1G and S3B). Similar effects on the protein expressions above were also observed following treatment with BAY-1143572 in SET-2 and HEL92.1.7 cells (Fig. S3C). These findings underscore that, following treatment with CDK9i, the repression of pro-growth and anti-apoptotic proteins observed could contribute to lowering of the apoptotic threshold and loss of viability of post-MPN sAML cells. We next determined in vivo activity of BAY-1143572 against HEL92.1.7 cells transduced with, and expressing, luciferase, following their infusion via tail vein and engraftment in the immune-depleted NSG mice. After engraftment of HEL92.1.7 cells, mice were treated by oral gavage with 10 mg/kg of BAY-1143572, or with the vehicle control, for 3 weeks. The route of administration and dose of BAY-1143572 chosen was previously shown to be safe in tumor xenograft studies [8]. As shown in Fig. 2A, and S4A, after only 2-weeks of treatment with BAY-1143572, there was significant reduction in sAML burden, whereas 3-weeks of treatment significantly improved the median and overall survival of the NSG mice, without any notable toxicity (Fig. 2B and S4B). We have previously interrogated and reported that as compared to their activity as monotherapy, targeted agents against epigenetic regulators, including inhibitor of BETP, KDM1A (LSD1), EZH2 or HDACs, are significantly more effective when combined with ruxolitinib or an inhibitor of anti-apoptotic protein such as BCL2 and Bcl-xL [11, 12]. Therefore, we next determined whether co-treatment with different doses of CDK9i and ruxolitinib or of the BCL2/Bcl-xL inhibitor navitoclax (ABT-263) would exert synergistic in vitro lethality against post-MPN sAML cells, as determined by combination index (CI) median effect analysis of Chou and Talalay (CI < 1.0) [13, 14]. As monotherapy, navitoclax exhibited lethal activity against JAKi-sensitive SET-2 and HEL cells, SET-2-RuxP and HEL-RuxP cells as well as CD34+ post-MPN sAML cells but not against CD34+ normal progenitor cells (Fig. 2C, D and S4C), whereas ruxolitinib exerted modest lethality in PD, CD34+ post MPN sAML cells, it lacked any activity against CD34+ normal progenitors (Fig. S4D, E). As shown in Fig. 2E, F, co-treatment at different dose levels of BAY-1143572 or NVP2 and ruxolitinib or navitoclax induced synergistic apoptosis in not only the JAKi-sensitive SET-2 but also JAKi-P/R SET-2-RuxP cells. This CDK9i-based combination with ruxolitinib or navitoclax also induced synergistic loss of viability in genetically profiled, PD, CD34+ post-MPN sAML cells (CI < 1.0) (Figs. S1A, 2G, H). Collectively, our findings demonstrate that targeted inhibition of CDK9 exerts lethal in vitro activity against JAKi-sensitive and JAKi-tolerant/resistant post-MPN sAML cells, as well as exerts significant in vivo efficacy against post-MPN sAML cells. This is likely due to CDK9i-mediated abrogation of the dysregulated transcriptome underpinning the aggressive biology and therapy resistance of the post-MPN sAML cells [15]. They also show that co-treatment with CDK9i and ruxolitinib or navitoclax exhibits synergistically lethal activity against not only JAKi-sensitive but also JAKi-P/R post-MPN sAML cells. Early phase clinical trials of monotherapy with CDK9is, e.g., NCT03263637 and NCT04630756, and of navitoclax, e.g., NCT04041050, are currently being conducted. Findings presented here merit, and may guide further, in vivo confirmation of efficacy of these CDK9i-based combinations in post-MPN sAML.

**Fig. 1:**
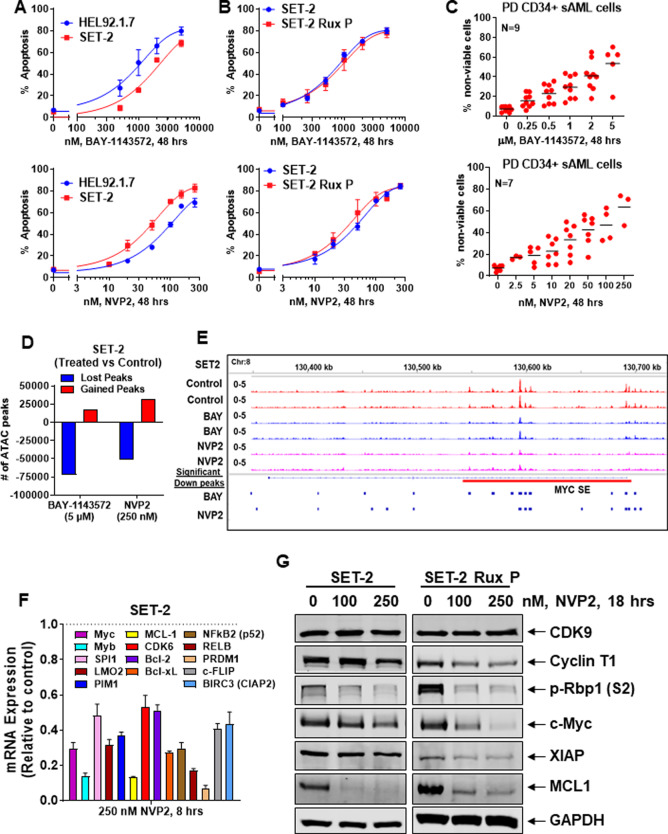
Treatment with CDK9 inhibitors dose-dependently induced apoptosis, decreased chromatin accessibility and depleted oncogene expressions in sAML cells. **A** HEL92.1.7 (HEL) and SET-2 cells were treated with the indicated concentrations of BAY-1143572 (BAY) or NVP2 for 48 h. At the end of treatment, cells were washed with 1× PBS and stained with annexin V and To-PRO-3 iodide. The % of annexin V-positive, apoptotic cells were determined by flow cytometry. Curves represent the mean of three experiments ± S.E.M. **B** SET-2 and ruxolitinib-persister/resistant SET-2-RuxP cells were treated with the indicated concentrations of BAY-1143572 or NVP2 for 48 h. Following this, the % of annexin V-positive, apoptotic cells were determined by flow cytometry. Curves represent the mean of three experiments ± S.E.M. **C** PD, CD34+ sAML cells were treated with the indicated concentrations of BAY-1143572 or NVP2 for 48 h. Following this, cells were washed with 1× PBS and stained with propidium iodide (PI). The % of PI-positive, non-viable cells were determined by flow cytometry. Horizontal black lines represent the mean loss of viability in the PD sAML samples. **D** SET-2 cells were treated with 5 µM of BAY-1143572 or 250 nM of NVP for 16 h. Total nuclei were isolated and ATAC-Seq analysis was performed utilizing Tn5 transposase and next generation sequencing. The total number of gained and lost peaks in the treated versus untreated cells was determined utilizing diffReps. **E** IGV plot of ATAC-Seq peak densities in SET-2 cells treated with BAY-1143572 or NVP2 for 16 h. Significantly altered down peaks (≥1.25-fold down relative to untreated and *p*-value <0.05) are noted by blue boxes. The red bar indicates the position of the MYC super enhancer locus (Enhancer regions 1–5). The approximate locations of the individual enhancer peaks are E1 = 130,548 kb; E2 = 130, 559 kb; E3 = 130, 595 kb; E4 = 130, 604 kb and E5 = 130, 679 kb. **F** SET-2 cells were treated with 250 nM of NVP2 for 8 h. Total RNA was harvested and utilized for reverse transcription. The resulting cDNA was utilized for quantitative PCR with TaqMan probes as indicated. The relative expression of each mRNA was normalized to the expression of GAPDH and compared to the untreated control cells. **G** Immunoblot analysis of SET-2 and SET-2-RuxP cells following 18 h of treatment with NVP2. The expression levels of GAPDH in the cell lysates served as the loading control.

**Fig. 2:**
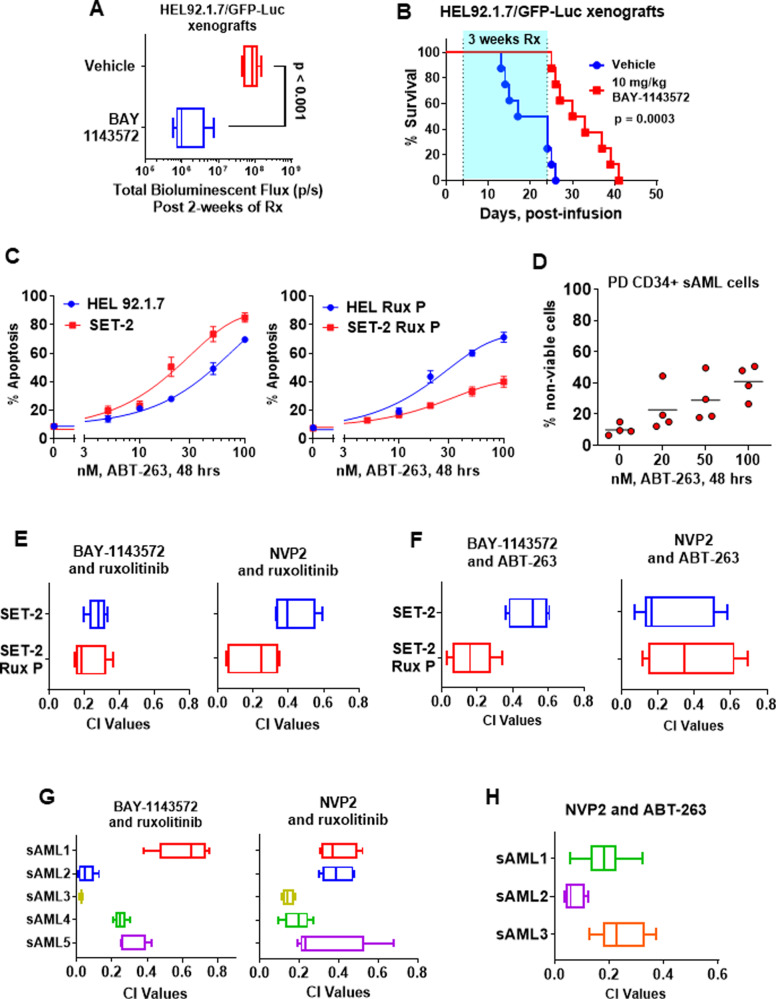
Treatment with CDK9 inhibitor markedly reduced leukemia burden, significantly improved the median and overall survival of NSG mice engrafted with luciferase-transduced sAML cells and exerted synergistic in vitro lethality with ruxolitinib or navitoclax (ABT-263). **A** Quantification of total bioluminescent flux (photons/second) in NSG mice engrafted with luciferase-expressing HEL92.1.7 cells and treated for two weeks with vehicle or 10 mg/kg of BAY-1143572. **B** Kaplan–Meier survival plot of NSG mice engrafted with luciferase-expressing HEL92.1.7 cells and treated for 3 weeks with vehicle or 10 mg/kg of BAY-1143572. **C** HEL92.1.7, SET-2, HEL-Rux P, and SET-2-Rux P cells were treated with the indicated concentrations of ABT-263 for 48 h. Following this, the % of annexin V-positive, apoptotic cells were determined by flow cytometry. Curves represent the mean of three experiments ± S.E.M. **D** PD, CD34+ sAML cells (*n* = 4) were treated with the indicated concentrations of ABT-263 for 48 h. Following this, the % of PI-positive, non-viable cells were determined by flow cytometry. **E**, **F** SET-2 and SET-2-Rux P cells were treated with BAY-1143572 or NVP2 and/or ruxolitinib or ABT-263 for 48 h. At the end of treatment, the % of annexin V-positive, apoptotic cells were determined by flow cytometry. The combination index (CI) values for each combination were calculated with CompuSyn and graphed with GraphPad V8. CI values less than 1.0 indicate a synergistic interaction of the combination. **G** PD, CD34+ sAML cells (*n* = 5) were treated with BAY-1143572 or NVP2 and/or ruxolitinib for 48 h. Following this, the % of PI-positive, non-viable cells were determined by flow cytometry. The combination index (CI) values for each combination were calculated with CompuSyn and graphed with GraphPad V8. CI values less than 1.0 indicate a synergistic interaction of the combination. **H** PD, CD34+ sAML cells (*n* = 3) were treated with NVP2 and/or ABT-263 for 48 h. Then, the % of PI-positive, non-viable cells were determined by flow cytometry. The combination index (CI) values for each combination were calculated with CompuSyn and graphed with GraphPad V8. CI values less than 1.0 indicate a synergistic interaction of the combination.

## Supplementary information

Supplemental Figures

Supplemental Figure Legends
