# Supplementary material for: Efficacy of CDK9 inhibition in therapy of post-myeloproliferative neoplasm (MPN) secondary (s) AML cells
Source: Blood Cancer J. 2022 Jan 31;12(1):23. doi: 10.1038/s41408-022-00618-4 (PMC8803998; doi:10.1038/s41408-022-00618-4)
Supplement: Supplementary file 1 — Supplemental Figures [file 41408_2022_618_MOESM1_ESM.pdf]

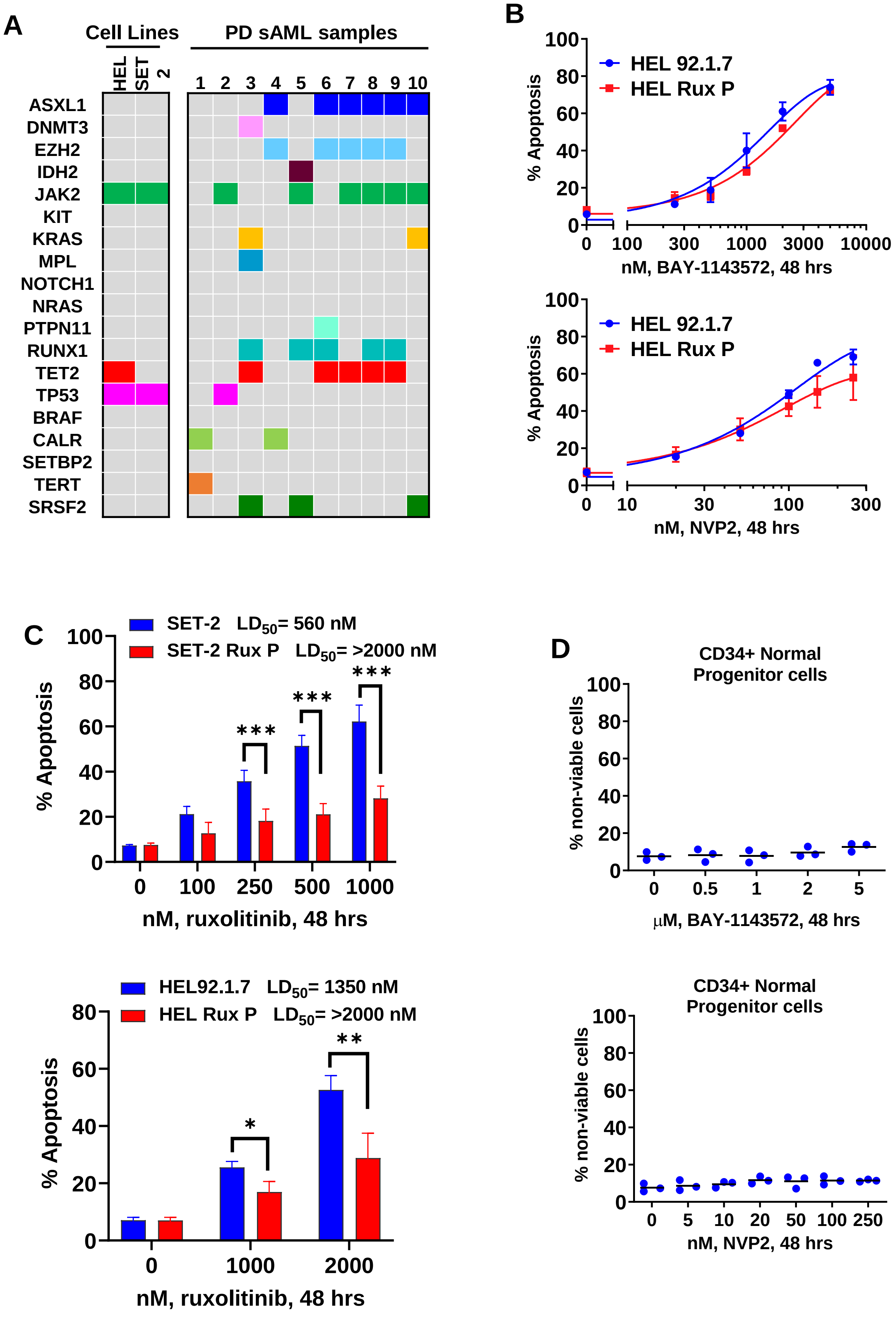
A
Cell Lines
PD sAML samples
HEL
SET 2
1 2 3 4 5 6 7 8 9 10
ASXL1
DNMT3
EZH2
IDH2
JAK2
KIT
KRAS
MPL
NOTCH1
NRAS
PTPN11
RUNX1
TET2
TP53
BRAF
CALR
SETBP2
TERT
SRSF2
B
% Apoptosis
HEL 92.1.7
HEL Rux P
0 100 300 1000 3000 10000
nM, BAY-1143572, 48 hrs
0 10 30 100 300
nM, NVP2, 48 hrs
C
SET-2 LD50= 560 nM
SET-2 Rux P LD50= >2000 nM
***
0 100 250 500 1000
nM, ruxolitinib, 48 hrs
HEL92.1.7 LD50= 1350 nM
HEL Rux P LD50= >2000 nM
*
**
0 1000 2000
nM, ruxolitinib, 48 hrs
D
CD34+ Normal Progenitor cells
% non-viable cells
0 0.5 1 2 5
μM, BAY-1143572, 48 hrs
0 5 10 20 50 100 250
nM, NVP2, 48 hrs

Figure S1

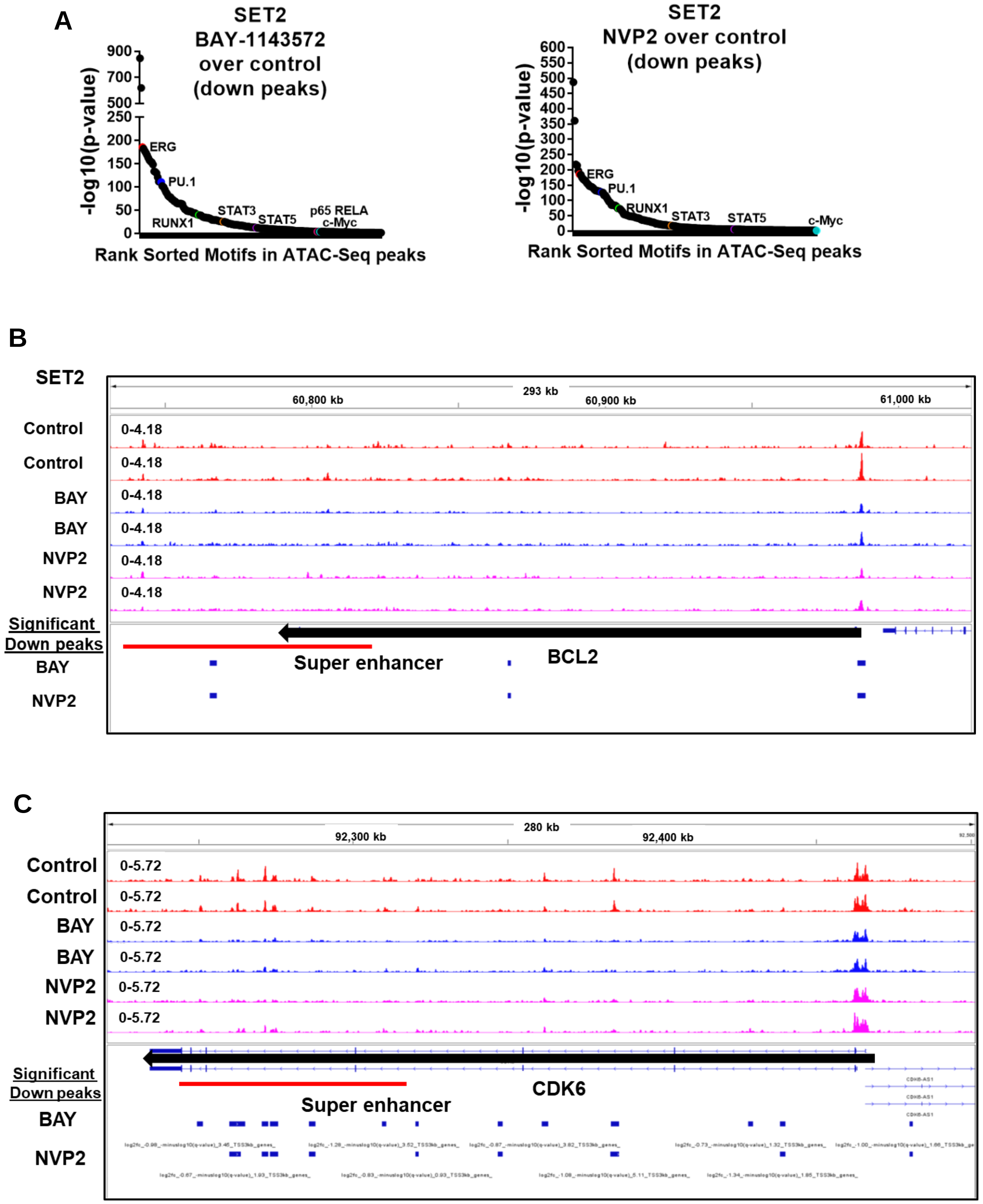
A
SET2
BAY-1143572
over control
(down peaks)
-log10(p-value)
900
700
500
250
200
150
100
50
0
ERG
PU.1
RUNX1
STAT3
STAT5
p65 RELA
c-Myc
Rank Sorted Motifs in ATAC-Seq peaks
SET2
NVP2 over control
(down peaks)
-log10(p-value)
600
550
500
450
400
350
300
250
200
150
100
50
0
ERG
PU.1
RUNX1
STAT3
STAT5
c-Myc
Rank Sorted Motifs in ATAC-Seq peaks
B
SET2
293 kb
60,800 kb
60,900 kb
61,000 kb
Control
0-4.18
Control
0-4.18
BAY
0-4.18
BAY
0-4.18
NVP2
0-4.18
NVP2
0-4.18
Significant
Down peaks
BAY
NVP2
Super enhancer
BCL2
C
280 kb
92,300 kb
92,400 kb
Control
0-5.72
Control
0-5.72
BAY
0-5.72
BAY
0-5.72
NVP2
0-5.72
NVP2
0-5.72
Significant
Down peaks
BAY
NVP2
Super enhancer
CDK6

Figure S2

D

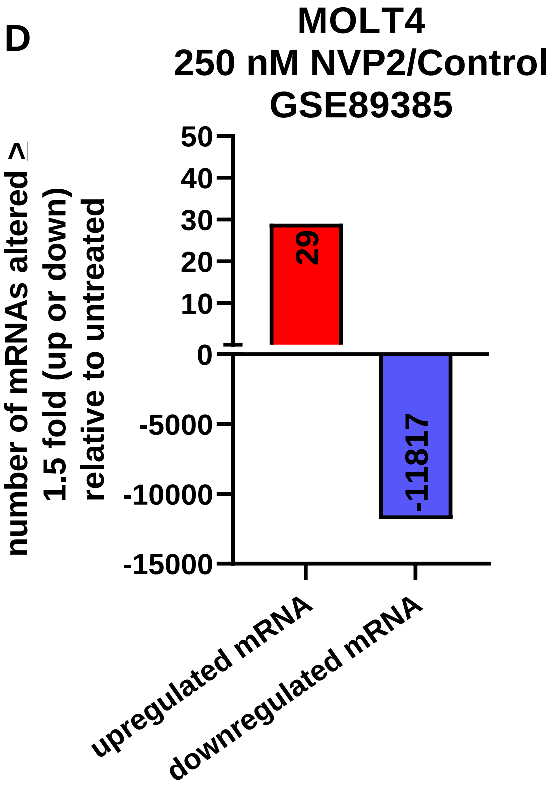
MOLT4
250 nM NVP2/Control
GSE89385
number of mRNAs altered ≥ 1.5 fold (up or down) relative to untreated
50
40
30
20
10
0
-5000
-10000
-15000
29
-11817
upregulated mRNA
downregulated mRNA

E

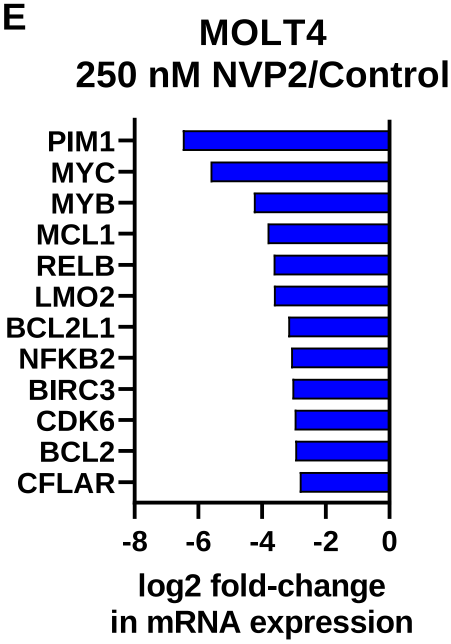
MOLT4
250 nM NVP2/Control
PIM1
MYC
MYB
MCL1
RELB
LMO2
BCL2L1
NFKB2
BIRC3
CDK6
BCL2
CFLAR
-8
-6
-4
-2
0
log2 fold-change in mRNA expression

Figure S2

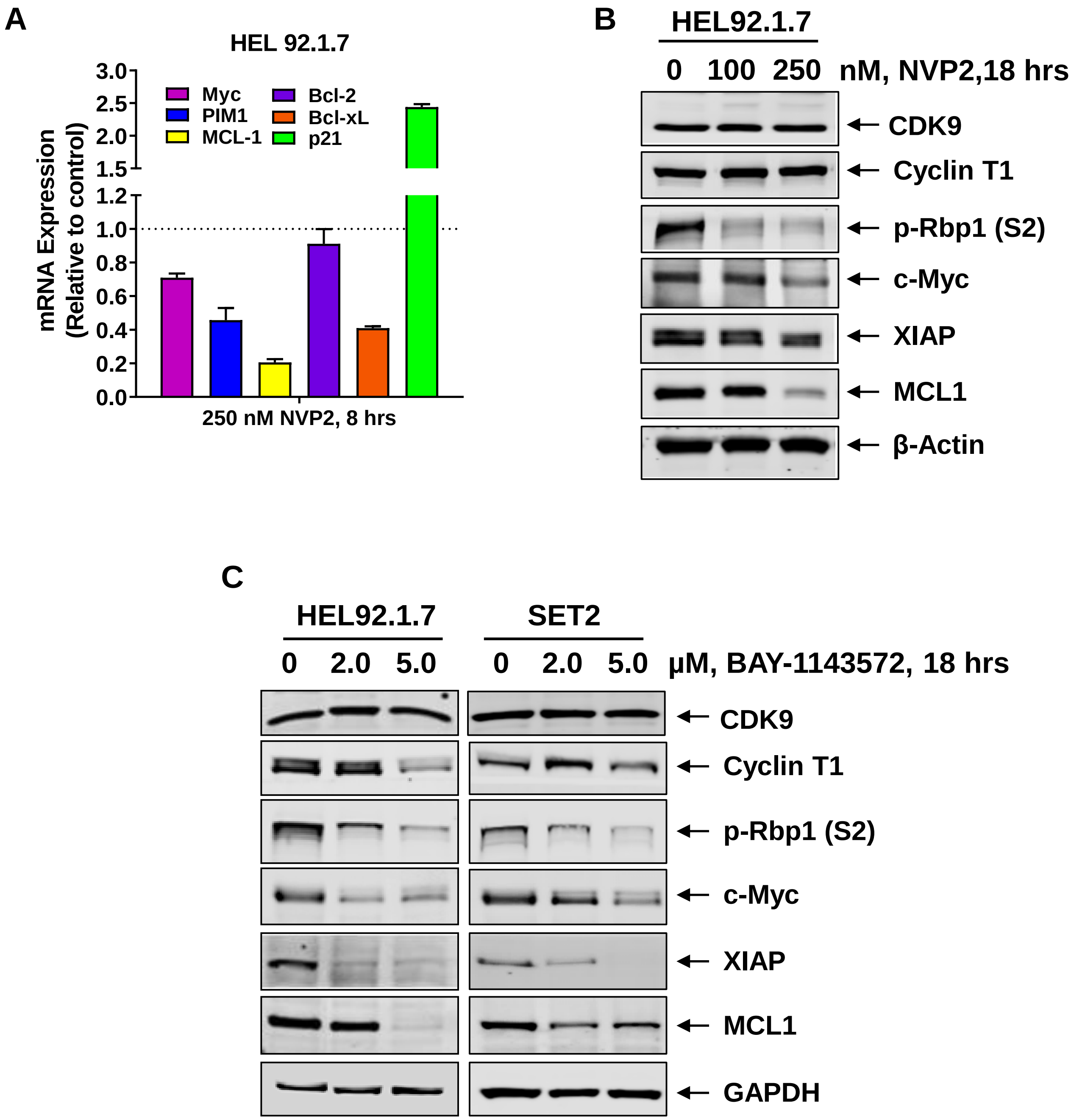
A
HEL 92.1.7
mRNA Expression
(Relative to control)
3.0
2.5
2.0
1.5
1.2
1.0
0.8
0.6
0.4
0.2
0.0
Myc
PIM1
MCL-1
Bcl-2
Bcl-xL
p21
250 nM NVP2, 8 hrs
B
HEL92.1.7
0 100 250 nM, NVP2,18 hrs
CDK9
Cyclin T1
p-Rbp1 (S2)
c-Myc
XIAP
MCL1
β-Actin
C
HEL92.1.7
SET2
0 2.0 5.0 0 2.0 5.0 µM, BAY-1143572, 18 hrs
CDK9
Cyclin T1
p-Rbp1 (S2)
c-Myc
XIAP
MCL1
GAPDH

Figure S3

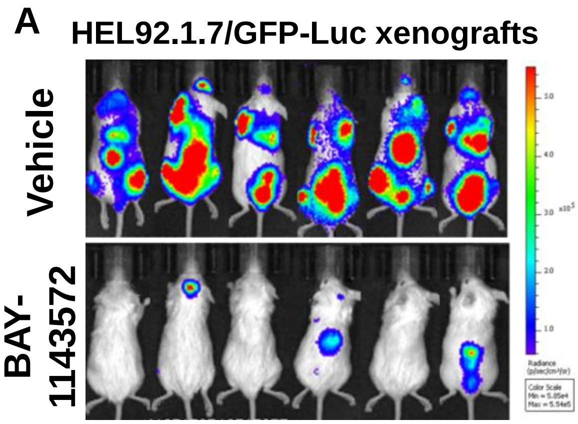

B

Treatment	Median Survival (Days)
Vehicle	20.5
10 mg/kg BAY-1143572	31.5

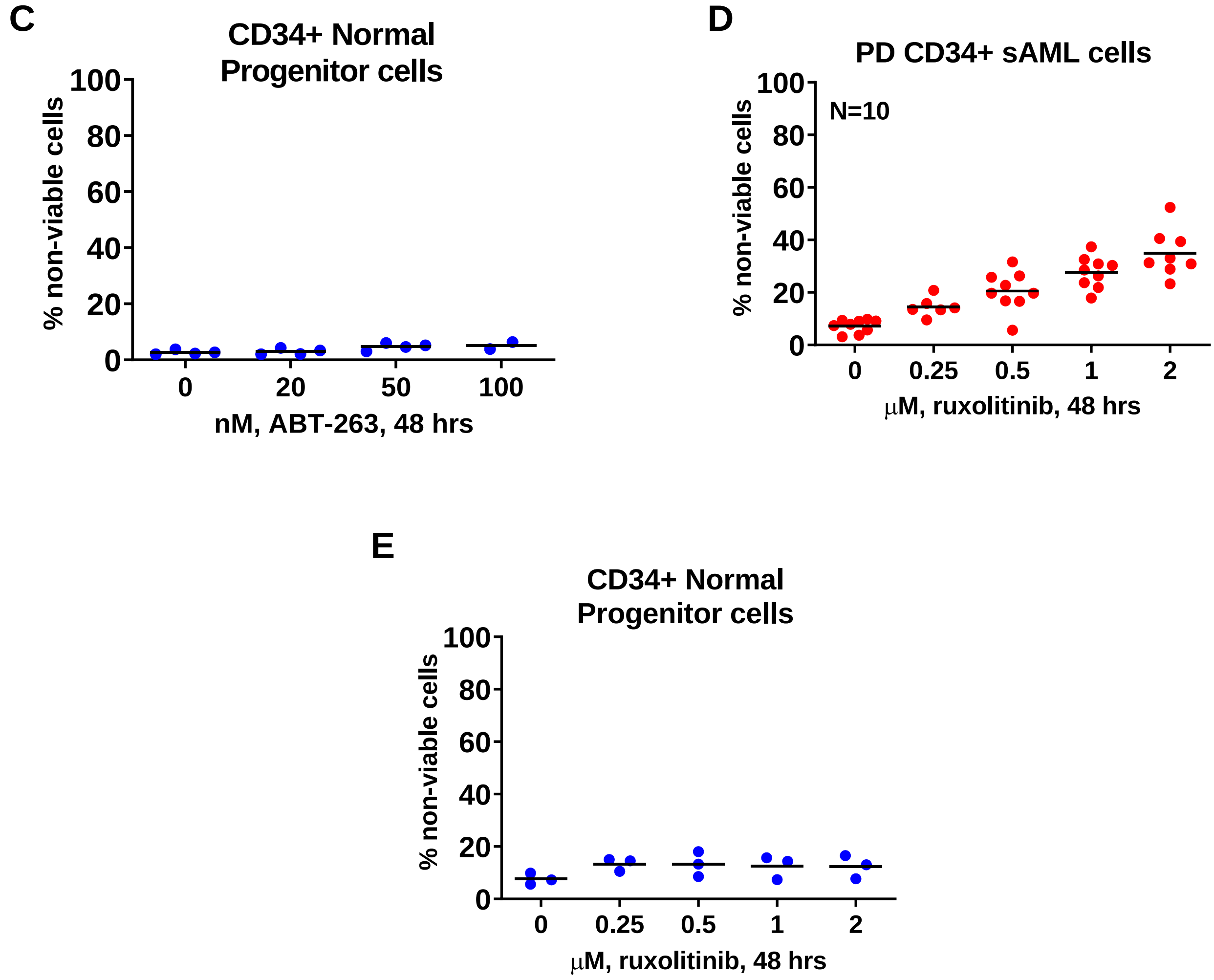

Figure S4
