# Supplementary material for: Efficacy of CDK9 inhibition in therapy of post-myeloproliferative neoplasm (MPN) secondary (s) AML cells
Source: Blood Cancer J. 2022 Jan 31;12(1):23. doi: 10.1038/s41408-022-00618-4 (PMC8803998; doi:10.1038/s41408-022-00618-4)
Supplement: Supplementary file 2 — Supplemental Figure Legends [file 41408_2022_618_MOESM2_ESM.docx]

**Supplemental Figure Legends**

**Supplemental Figure 1. Treatment with CDK9 inhibitor dose-dependently induced apoptosis of sAML HEL92.1.7 and ruxolitinib persister/resistant HEL-Rux P cells but exhibited minimal cytotoxicity against normal CD34+ hematopoietic progenitor cells (HPCs)**. **A**. Oncoplot of mutations (identified by NGS) in the patient-derived sAML cells utilized in these studies. **B**. HEL92.1.7 and ruxolitinib-persister/resistant HEL-RuxP cells were treated with the indicated concentrations of BAY-1143572 or NVP2 for 48 hours. Following this, the % of annexin V-positive, apoptotic cells were determined by flow cytometry. Curves represent the mean of three experiments + S.E.M. **C**. SET-2, SET-2 RuxP, HEL92.1.7 and HEL-RuxP cells were treated with the indicated concentrations of ruxolitinib for 48 hours. Following this, the % of annexin V-positive, apoptotic cells were determined by flow cytometry. Columns represent the mean of three experiments + S.E.M. The LD_50_ dose of ruxolitinib was calculated by GraphPad V8. * = p < 0.05; ** = p < 0.01; *** = p< 0.005 determined by two-tailed, unpaired, t-test in GraphPad V8. **D**. Normal CD34+ HPCs from cord blood were treated with the indicated concentrations of BAY-1143572 (upper panel) or NVP2 (lower panel) for 48 hours. Then, cells were washed with 1X PBS and stained with propidium iodide (PI). The % of PI-positive, non-viable cells were determined by flow cytometry.

**Supplemental Figure 2. Treatment with CDK9 inhibitors decreased chromatin accessibility and transcription factor binding site availability in sAML cells and decreased global mRNA expression**. **A**. SET-2 cells were treated with 5 µM of BAY-1143572 or 250 nM of NVP2 for 16 hours. Total nuclei were isolated and ATAC Seq analysis was performed utilizing Tn5 transposase. DNA fragments were indexed, libraries were prepared, pooled and next generation sequencing was performed. Rank-sorted transcription factor motif binding site changes in the lost (down) peaks in SET-2 cells treated with BAY or NVP2 for 16 hours were determined utilizing HOMER. The -log10 p-value for the changes in transcription factor binding sites is shown. Notable sAML-relevant TF motif alterations are labeled. **B-C**. IGV plots of ATAC-Seq peak densities in SET-2 cells treated with BAY-1143572 or NVP2 for 16 hours. Black arrows indicate the orientation of the reading frame for the gene. Locations of significantly altered down peaks (> 1.25-fold decreased relative to untreated and p-value < 0.05) are noted by blue boxes. The red bars indicate the position of the BCL2 super enhancer and the CDK6 super enhancer, respectively. **D**. We utilized publicly available RNA Seq data from dataset GSE89385 to observe the effects of CDK9 inhibition on global mRNA expression. Panel shows the total mRNA expression alterations (>1.5 fold up or down relative to untreated cells) identified by RNA-Seq analysis in MOLT4 cells treated with 250 nM of NVP2 for 6 hours. **E**. Log2 fold-change in selected mRNAs from MOLT4 cells treated with 250 nM of NVP2 for 6 hours (dataset GSE89385).

**Supplemental Figure 3**. **Treatment with CDK9 inhibitor BAY-1143572 depleted sAML relevant oncogene expressions in SET-2 and HEL92.1.7 cells**. **A**. HEL92.1.7 cells were treated with 250 nM of NVP2 for 8 hours. Total RNA was harvested and utilized for reverse transcription. The resulting cDNA was analyzed by quantitative PCR with TaqMan probes as indicated. The relative expression of each mRNA is normalized to the expression of GAPDH and compared to the untreated control cells. **B**. Immunoblot analysis of HEL92.1.7 cells following 18 hours of treatment with NVP2. The expression levels of β-Actin in the cell lysates served as the loading control. **C**. Immunoblot analysis of HEL92.1.7 and SET-2 cells following 18 hours of treatment with the indicated concentrations of BAY-1143572. The expression levels of GAPDH in the cell lysates served as the loading control.

**Supplemental Figure 4**. **Treatment with ruxolitinib dose-dependently induced loss of viability of patient-derived, CD34+ sAML cells while sparing normal CD34+ HPCs.**  **A**. Bioluminescent imaging of NSG mice engrafted with luciferase-expressing HEL92.1.7 cells and treated for two weeks with vehicle or 10 mg/kg of BAY-1143572 (P.O. by oral gavage, daily x 5 days per week). **B**. Median survival of NSG mice engrafted with luciferase-expressing HEL92.1.7 cells and treated for three weeks with vehicle or 10 mg/kg of BAY-1143572. **C**. Normal CD34+ cord blood progenitor cells were treated with the indicated concentrations of ABT-263 for 48 hours. Following this, the % of PI-positive, non-viable cells were determined by flow cytometry. **D-E**. PD, CD34+ sAML cells from patients (n=10) and normal CD34+ hematopoietic progenitor cells (n=3) were treated with the indicated concentrations of ruxolitinib for 48 hours. Following this, the % of PI-positive, non-viable cells were determined by flow cytometry.
